# Dermatoscopy of cellular neurothekeoma

**DOI:** 10.1016/j.jdcr.2022.01.036

**Published:** 2022-02-08

**Authors:** Paolo Bortoluzzi, Maurizio Romagnuolo, Pier Luca Mandolini, Emilio Berti, Francesca Boggio

**Affiliations:** a Postgraduate School of Dermatology and Venereology, Università degli Studi, Milan, Italy; b Dermatology Unit, Fondazione IRCCS Ca' Granda Ospedale Maggiore Policlinico, Milan, Italy; c Department of Pathophysiology and Transplantation, Università degli Studi di Milano, Milan, Italy; d Division of Pathology, Fondazione IRCCS Ca' Granda Ospedale Maggiore Policlinico, Milan, Italy

**Keywords:** cellular neurothekeoma, dermatoscopy, histopathology, CN, cellular neurothekeoma

## Clinical presentation

**Fig 1:**
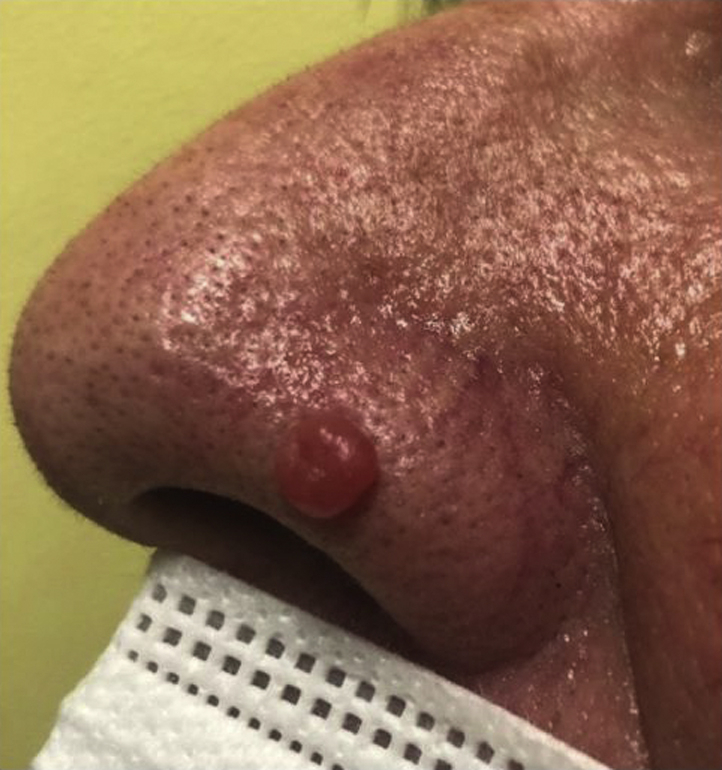
A red and soft-at-palpation papule on the nasal tip with a diameter of approximately 5 mm.

A 68-year–old woman presented with a 7-month history of an asymptomatic, slow-growing lesion on the nasal tip. Physical examination revealed a red and soft-at-palpation papule on the nasal tip, with a diameter of approximately 5 mm (Fig 1).

## Dermatoscopic apparearance

**Fig 2:**
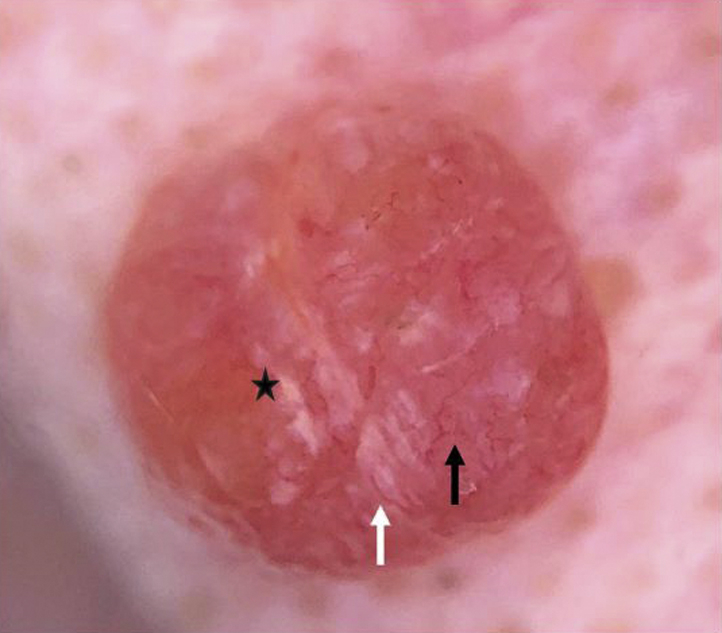
Contact nonpolarized dermatoscopic image showing arborizing vessels (*black arrow*), linear vessels (*white arrow*), and whitish streak areas (*black star*) on an erythematous-orange structureless background. (Original magnification: ×10.)

Contact nonpolarized dermatoscopic examination revealed a nonspecific pattern with irregular linear vessels, arborizing vessels, and whitish streak areas on an erythematous-orange structureless background (Fig 2).

## Histologic diagnosis

**Fig 3:**
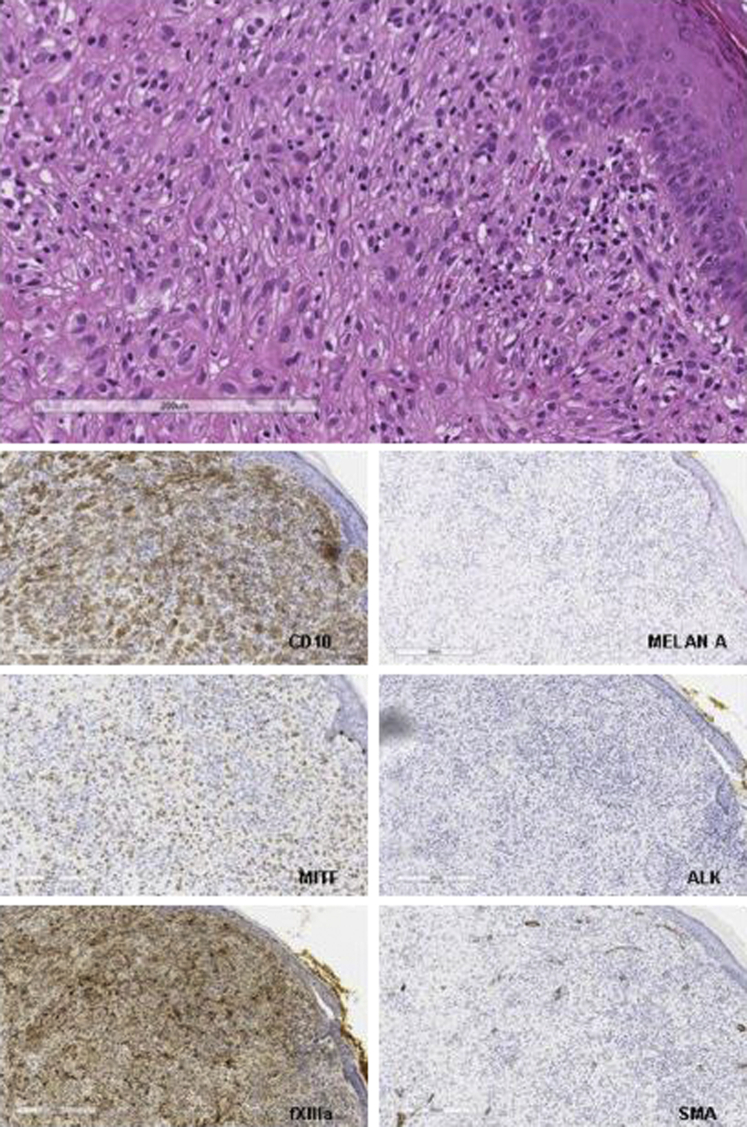
Cellular neurothekeoma (hematoxylin-eosin stain; original magnification: ×20). Dermal proliferation of epithelioid cells in small nests with no connection to the epidermis. The neoplastic cells were positive (on the left side, from top to bottom) for CD10, MITF, and factor XIIa (fXIIIa) and negative (on the right side, from top to bottom) for Melan A, ALK, and smooth muscle actin.

The lesion consisted of a nodular dermal circumscribed proliferation with lobular growth, uninvolving the epidermis and composed by round epithelioid cells with a vesicular nucleus and small nucleoli arranged in small nests. No mitotic features were observed. Immunohistochemically, the lesion expressed CD10, factor XIIIa, MITF, and CD68 (focally). All melanocytic and neural marker tests were negative. Moreover, the lesion was negative for smooth muscle actin, EMA, CD163, and ALK. Proliferating index Ki67 was established to be about 2% to 3% (Fig 3).

Considering the histologic features together with immunohistochemical profile, a diagnosis of cellular neurothekeoma (CN) was reached.

**Key message**

CN is a rare, benign, cutaneous tumor probably originating from fibroblastic cells that differentiate into myofibroblasts and recruit histiocytes.^1^ It usually presents in young women as a solitary, asymptomatic, low-growing, erythematous-to-brownish papule or nodule on the head and neck area or on the upper extremities. Dermatoscopic diagnosis is challenging, and it is often mistaken for basal cell carcinoma because of the characteristics of arborizing vessels. Unlike basal cell carcinoma, which presents crystalline structures and shiny white streaks with polarized dermatoscopy, CN is characterized by whitish structures on contact nonpolarized dermatoscopy that correspond to peripheral fibrosis and fibrous septa.^2^ These dermatoscopic features could help physicians differentiate between these 2 types of tumors. The diagnosis of CN usually requires histopathologic confirmation.

## Conflicts of interest

None disclosed.
